# Gait adaptations in step length and push-off force during walking with functional asymmetry

**DOI:** 10.3389/fbioe.2025.1550710

**Published:** 2025-03-28

**Authors:** Tomislav Baček, Yufan Xu, Liuhua Peng, Denny Oetomo, Ying Tan

**Affiliations:** ^1^ School of Electrical, Mechanical and Infrastructure Engineering Faculty of Engineering and Information Technology, The University of Melbourne, Parkville, VIC, Australia; ^2^ Faculty of Science, The University of Melbourne, Parkville, VIC, Australia

**Keywords:** functional gait asymmetry, statistical gait modelling, step length adaptations, push-off force dynamics, cross-dataset gait predictions

## Abstract

Human walking is highly adaptable, allowing individuals to maintain efficiency and stability across diverse conditions. However, how gait adapts to functional asymmetry remains poorly understood. This study addresses this gap by employing a within-subject design to isolate the effect of functional asymmetry using a unilateral knee constraint to emulate hemiparetic gait. This approach eliminates inter-individual variability present in previous studies. A dataset of 19 participants walking across 30 conditions was used to examine these adaptations in step length and push-off force in both absolute terms and symmetry metrics. Results reveal that functional asymmetry disproportionately impacts propulsion, with constrained-leg force decreasing significantly at higher speed, while step length symmetry remains stable. This suggests a prioritisation of spatial over kinetic symmetry, likely to optimise walking energetics and maintain anterior-posterior balance. Statistical models demonstrated good within-dataset performance but limited generalisability across dataset predictions, emphasising the challenges of applying models across studies of different designs. These findings highlight critical limitations in applying statistical models trained on healthy persons to patient populations and provide insights into key biomechanical adaptations that could inform individualised biofeedback strategies for hemiparetic patients. Understanding individual compensations for unilateral deficits could help refine rehabilitation interventions that target propulsion deficits and optimise gait symmetry.

## 1 Introduction

Human walking is remarkably versatile, driven by complex adaptation mechanisms that allow individuals to maintain efficiency, stability, and control in a variety of conditions. Healthy individuals generally prefer to walk at a speed that minimises energy expenditure per unit of distance traveled (Ralston, 1958; Molen et al., 1972). To achieve this, they actively adjust gait variables, including step width (Donelan et al., 2001), step length (Minetti et al., 1995), step time (Ellis et al., 2013), and push-off force (Reimann et al., 2018), while avoiding penalties associated with asymmetries (Ellis et al., 2013). These adjustments vary with walking speed: changes in step width support lateral balance (Orendurff et al., 2004; Bruijn and Dieen, 2018), while changes in step length accommodate the increased demands of push-off (Donelan et al., 2002; Yanez et al., 2023), all while maintaining a sufficient margin of stability (Hak et al., 2013a). This adaptability allows people to seamlessly walk on slopes (Franz and Kram, 2012; Xie and Chien, 2024), in the presence of lateral perturbations (Frame et al., 2020; Bruijn and Dieen, 2018), or with physical changes of aging (Owings and Grabiner, 2004; Dean et al., 2007).

In contrast, hemiparetic individuals walk with slower speeds (Brandstater et al., 1983; Olney and Richards, 1996) and higher energetic cost (Stoquart et al., 2012) while exhibiting spatio-temporal asymmetries. These include the extended stance phase on the non-paretic side, prolonged swing phase on the paretic side (Kim and Eng, 2003), and reduced paretic propulsion (Bowden et al., 2006). The said asymmetries manifest in both spatio-temporal asymmetry (Wall and Turnbull, 1986; Hsu et al., 2003; Patterson et al., 2010) and asymmetries in push-off force and impulse (Lewek et al., 2018; Padmanabhan et al., 2020). Consequently, some patients take longer paretic steps while others take shorter ones (Kim and Eng, 2003). However, despite these differences and additional effort they need to exert to adapt, hemiparetic individuals can still independently adjust gait parameters, relying on strategies that include increased non-paretic propulsion or wider steps (Hak et al., 2013b; Lewek et al., 2014) to maintain stability and forward progression.

While much is known about gait adaptations in both healthy and patients, significant gaps remain. Many studies rely on speed-matching or age-matching healthy and patient groups, which fails to account for intrinsic biomechanical differences (Lim et al., 2022; Booij et al., 2021). Such approaches can mask critical insights by overlooking how baseline differences, such as muscle weakness, pain, or comorbidities, alter gait dynamics independently of age or walking speed. Moreover, walking speed alone is a limited indicator of gait function and recovery, providing an incomplete picture of metrics like step length and forward propulsion (Roelker et al., 2019). These limitations challenge the validity of using matched-group analyses or statistical models trained in healthy populations to explain or predict patient gait, as such models tend to overfit dataset-specific dynamics.

To address this gap, this study investigates how healthy individuals adapt their gait parameters across varying speeds and cadences under imposed functional asymmetry (i.e., *a difference in the number of freely moving joints or their available range of motion between the two legs*). Specifically, we leverage a within-subject design and a unilateral passive knee constraint—two key methodological features of the dataset described in (Baček et al., 2024). The within-subject design ensures that each participant serves as their own control, isolating the mechanical effects of functional asymmetry on step length and push-off force (forward propulsion) without the influence of neurological deficits common in patient populations. The unilateral knee constraint serves as a model for hemiparetic gait by restricting sagittal-plane knee movement, emulating the reduced knee flexion observed in stroke survivors (Ali et al., 2014). While this approach does not replicate neurological factors found in hemiparetic patients, such as spasticity, proprioceptive deficits, or altered neuromuscular control, it provides a controlled experimental setting for analysing compensatory gait adaptations. By systematically studying these adaptations, this work offers insights into rehabilitation strategies that target propulsion deficits and gait symmetry in stroke patients.

Step length and push-off force are key to understanding anterior-posterior balance (Bruijn and Dieen, 2018) and the metabolic and mechanical costs of walking (Donelan et al., 2002). These parameters are increasingly used as biofeedback targets in hemiparetic rehabilitation (Yanez et al., 2023), making it crucial to understand their interplay and adaptability across conditions. In studying this interplay, this work addresses three key research questions: (i) How does a unilateral knee constraint affect step length and push-off force at different walking speeds? (ii) Do individuals prioritise kinetic (propulsion) or spatial (step length) symmetry when adapting to asymmetry? (iii) To what extent can statistical models trained in free-walking data predict gait adaptations under constrained conditions, and what are the limitations of applying these models to clinical populations? Through this systematic analysis, this study provides new insights into gait compensation mechanisms, with potential applications in individualised biofeedback-based gait rehabilitation for stroke patients.

## 2 Materials and methods

To address the three research questions, we analysed walking data from a publicly available dataset that includes both free and constrained walking conditions. The following section details the experimental design, data processing and analysis methods, and the definitions of key gait metrics examined.

### 2.1 Walking dataset

A publicly available dataset from (Baček et al., 2023) was used to investigate the effects of functional gait asymmetry on step length (SL) and peak push-off force (PO) in this paper. We refer to this dataset as B24. In this context, functional asymmetry refers to a constrained condition in which a knee brace was used to unilaterally restrict knee joint movement. The dataset comprises data from 21 neurotypical young adults (age 30 ± 8 years, body mass 72.7 ± 12.3 kg, height 1.72 ± 0.09 m), all of whom were free of any lower-extremity injury and wore low-profile shoes during the trials. Two participants (Subjects 7 and 12 in the original dataset) only completed the first data collection session and were excluded from this analysis. No data were missing for the remaining 19 participants analysed in this paper. The anthropometric characteristics of participants in the B24 dataset are given in the Appendix (Supplementary Table A1). The human study from (Baček et al., 2024) used in this analysis was approved by the ethics committee of The University of Melbourne, where the study was conducted (reference number: 2021-20623-13486-3).

The trials were conducted on a dual-belt instrumented treadmill, with each lasting 5 min. Participants completed a total of 30 trials: 15 without any constraints (hereafter referred to as *Free*) and 15 with their left knee joint fully extended via a passive knee brace (hereafter referred to as *Constrained*). In both conditions, participants walked at three speeds (0.4, 0.8, and 1.1 m/s) and five step frequencies peer speed, guided by a metronome (90%, 95%, 100%, 110%, and 120% of the preferred cadence, presented in random order). The 30 trials took place across two sessions on separate days, with 15 trials per day. The five cadences at each speed were organised into a continuous 25-min walking bout with no breaks between cadences. Bouts were arranged such that no two consecutive bouts involved the same walking conditions (either *Free* or *Constrained*) within a session, and all three speeds were included in each session. Marker data were collected at 100 Hz and ground reaction force (GRF) data at 1,000 Hz. Supplementary Figure A9 in Appendix gives an overview of the study design; a detailed description of the study is provided in (Baček et al., 2024).

The knee joint orthosis was designed in-house and consisted of two 3D-printed cuffs–one for the thigh and one for the shank–connected by two metal bars via a double-hinge joint. The cuffs were available in multiple sizes and could slide along the bars, allowing for quick manual adjustments to optimise fit and comfort relative to the participant’s knee joint. Each cuff was secured with two Boa straps, ensuring stability, and the orthosis was always worn on the left leg. To replicate the restricted knee flexion commonly observed in hemiparetic gait, the orthosis was locked in full extension (corresponding to a biological knee joint angle of 0°) using bolts at the hinge joints. However, due to soft tissue compliance, some residual knee flexion was observed across participants. The maximum recorded knee flexion angle during the swing phase at 1.1 m/s reached 15°, compared to the typical unconstrained knee flexion of 75° during swing. Supplementary Figure A10 in Appendix gives an overview of the study design; more detailed description and visualisation of the knee orthosis can be found in (Baček et al., 2024).

For this analysis, we separated the B24 dataset into four groups, corresponding to the left and right legs in each of the two conditions (*Free* and *Constrained*). We refer to the data from the left leg during unconstrained walking as *Free Left* and the data from the right leg as *Free Right*. Similarly, *Constrained Left* represents data from the left leg during constrained walking and *Constrained Right* refers to the data from the right leg during constrained walking. Note that it is always the left leg that is constrained during all *Constrained* walking in the experiment. Hence, *Constrained Right* is the data of the unconstrained right leg in the *Constrained* walking condition. Given the strong correlation of gait parameters between *Free Left* and *Free Right*, we use *Free Left* as the baseline for the *Free* condition.

### 2.2 Data processing and analysis

All data processing–including filtering, segmenting, and grouping–was performed using custom-written scripts in Matlab 2024a. Raw GRF data were filtered using a low-pass Butterworth filter with a 6 Hz cut-off frequency (Winter 2009). The vertical component of the GRF signal was used to segment data into gait cycles, with a threshold set at 5% of the peak amplitude (e.g., for a 75 kg person, the threshold would be 75×9. 81×0.05 = 37 N). In our analysis, the same number of cycles was used for both legs, and all gait metrics represent an average over the last 60 s of each 5-min test.

Statistical modelling was done in Python 3.10.10 using *statsmodels*, *scipy*, and *numpy* packages, with a Linear Mixed Models (LMM) approach (Lindstrom and Bates, 1988). Each participant’s data was treated as a distinct group to account for constant anthropometric variables within each group while allowing variations in their gait metrics. Data from the left and right legs were modelled separately, treating peak push-off force (PO) and step length (SL) as response variables (model outputs), and walking speed, its square, and anthropometric data (sex, age, mass, leg length) as explanatory variables (model inputs). To account for multiple cadences per speed—a characteristic of the used dataset—we added additional model input to each model. In the case of PO model, we added cadence as this is the dataset’s defining feature and the leading difference between conditions at the same walking speed. In the case of SL model, we could not add cadence due to its direct relationship with speed and SL; instead, we added trailing limb angle (TA) due to its direct relationship with push-off force (Lewek et al., 2018). Both PO and SL models include a fixed effect for speed given the known relationship between the speed and the two response variables (Fukuchi et al., 2019a). Models also include a quadratic effect of speed, similar to the models in (Yanez et al., 2023), to account for non-linear relationship between speed and PO and SL, and a fixed intercept. In addition to these fixed effects, both models include a random slope for speed to account for the repeated measures structure of the B24 dataset and variability across participants. We report the best performing PO and SL models with speed variance, reflecting the average individual effect of speed.

Model estimation quality across combinations of independent variables (model inputs) was assessed using the Akaike Information Criterion (AIC), and we report AIC values for all models, along with the parameters for the best model for each model output. From the list of all models produced, we select ones with the lowest AIC values as final prediction models for those are the models of the highest estimation quality. We evaluate the predictive model performance using several complementary metrics, including R-Squared ( ${\text{R}}^{2}$ ), Mean Absolute Error (MAE), and Mean Absolute Percentage Error (MAPE)^1^. The accuracy of the predictive model was evaluated on the B24 dataset and further tested on an independent, publicly available dataset by (Fukuchi et al., 2018a; Fukuchi et al., 2018b) (details in Appendix).

Statistical data analyses were conducted in Python 3.10.10 using *scipy* package. The effects of speed, cadence, and condition (free vs constrained) on gait variables were assessed using two-way repeated measures ANOVA (RMANOVA) at a significance level of p = 0.05. Where statistically significant differences were found, pairwise *post hoc* analyses were carried out using Tukey’s honestly significant difference (HSD) test, with Bonferroni corrections applied. We categorise statistical significance as follows: weak significance (0.01 <p≤ 0.05), moderate significance (0.001 <p≤ 0.01), and strong significance ( p≤ 0.001).

### 2.3 Gait metrics

A gait cycle is defined as the time between two successive heel strikes of the same leg. Peak push-off force (PO) represents the maximum amplitude of the fore-aft component of the GRF during the stance phase, which spans from heel strike to toe-off of the same leg. Step length (SL) is the fore-aft distance between the two calcaneous (heel) markers at the time of the leading leg’s heel strike. Symmetry is defined as the percentage ratio between the two legs: left vs right for PO (100% indicating symmetry) and left vs left plus right for SL (50% indicating symmetry).

Trailing limb angle (TA) is defined as the maximum hip extension angle. The hip joint angle trajectory was calculated following the methods outlined in Research Methods in Biomechanics (Robertson et al., 2013) and according to International Society of Biomechanics (ISB) guidelines (Wu et al., 2002). Leg length was measured as the distance from the anterior superior iliac spine (ASIS) to the ipsilateral medial malleolus during static calibration in a standing position.

## 3 Results

### 3.1 Statistical modelling (absolute gait metrics)

#### 3.1.1 Model estimation quality

We trained models for peak push-off force (PO) and step length (SL) using combinations of walking speed and its square ( v and $v^{2}$ ) along with anthropometric variables (age, sex, mass, and leg Length). Additionally, we included step frequency (f) for the PO model and trailing limb angle (TA) for the SL model (using speed and cadence as SL model inputs would not be an estimate but a closed form solution due to SL=v/f ). We define three model types: *Model1*, trained with v and $v^{2}$ only; *Model2*, trained with v , $v^{2}$ , and either f or TA; and *Model3*, trained with v , $v^{2}$ , either f or TA, and all four anthropometric variables. Models incorporating individual anthropometric variable are not presented here, as analysis indicated that no single variable consistently achieved significance across multiple models.

Table 1 provides Akaike Information Criterion (AIC) values for both model outputs (PO, SL), including all three model levels and four data groups. As the table shows, walking speed and either cadence (for PO model) or TA (for SL model) were the only model inputs that significantly improved model estimation quality (as indicated by low AIC values), while adding anthropometric variables as model inputs had minimal to no impact. For this reason, all further modelling of PO and SL is performed using *Model2*. Table 2 presents non-standardised weights of each model variable for the best overall model (*Model3*), specifically for the *Free Right* condition in the case of PO and the *Constrained Left* condition for SL. This is useful as it allows real-world interpretability of the effect of each model variable. For example, the speed coefficient (weight) in PO model corresponds to the change in peak push-off force in [N/kg] for a 1 [m/s] increase in speed. To understand the relative importance of each model variable, we also present standardised weights in Table 3. To do this, we calculated z-score for peak push-off force, step length, and continuous predictors to standardise them; sex is a categorical variable, so it does not need to be standardised. A more detailed analysis of model estimation quality can be found in the Appendix.

**TABLE 1:** AIC values [−] for PO and SL model estimation *(Lower values mean better quality model)*.

Data group	Push-off force (PO)			Step length (SL)		
	Model 1	Model 2	Model 3	Model 1	Model 2	Model 3
Free Left	−162.18	−260.44	−270.64	−782.61	−1008.58	−1036.79
Free Right	−192.03	−270.77	−283.26	−752.03	−958.57	−985.08
Constrained Left	−13.265	−94.35	−94.11	−709.44	−1036.96	−1055.63
Constrained Right	−93.86	−175.76	−182.54	−746.34	−853.99	−870.60

**TABLE 2:** Non-standardised weights for the model that best fit PO and SL overall (*Model3*).

	Intercept [N/kg] or [m]	v [m/s]	v^2^ [m/s]^2^	Cadence [steps/min] or TA [deg]	Leg length [m]	Sex [M=0 or F=1]	Age [years]	Mass [kg]	v variance [m/s]
PO [N/kg]	0.84	1.668	0.338	−0.008	−0.417	0.036	0.006	−0.002	0.026
SL [m]	−0.197	0.74	−0.332	−0.012	0.201	0.03	0.003	−0.001	0.004

Cadence only used in PO model, and TA only in SL model. Weights are non-standardised.

**TABLE 3:** Standardised weights for the model that best fit PO and SL overall (*Model3*).

	Intercept	v	v^2^	Cadence or TA	Leg length	Sex	Age	Mass	v variance
PO	0.026	1.035	0.208	−0.413	−0.085	−0.099	0.055	−0.094	0.017
SL	−0.094	1.595	−1.027	−0.482	0.162	0.357	−0.015	0.086	0.017

Cadence only used in PO model, and TA only in SL model. Weights are standardised.

#### 3.1.2 Model prediction quality

**FIGURE 1:**
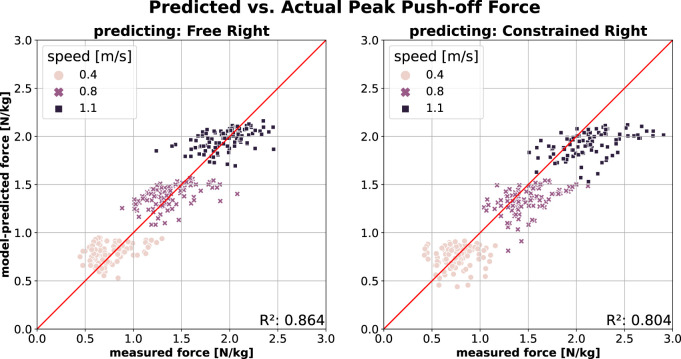
PO prediction of contralateral (i.e., right) leg using model trained on *Free Left* walking (*Model2*; model inputs: v , $v^{2}$ , f ). Right leg is unconstrained in both cases (Left) Predicting right leg’s PO in *Free* walking (Right) Predicting right leg’s PO in *Constrained* walking.

Figure 1 illustrates the prediction of PO using a model trained on the *Free Left* data (left leg during free walking). The model accurately predicts PO of the contralateral (right) leg in free walking across all three speeds (*Free Right*; Figure 1, left), achieving a Mean Absolute Error (MAE) of 0.16 N/kg and a Mean Absolute Percentage Error (MAPE) of 14.3%. However, prediction accuracy decreases when applied to constrained walking (*Constrained Right*; Figure 1, right), particularly at higher walking speeds, where the model sometimes underestimates the measured force. This is reflected in lower ${\text{R}}^{2}$ and higher errors (MAE = 0.21 N/kg and MAPE = 15.5%) compared to the *Free* condition. Notably, the right leg remains unconstrained in both cases, showing minimal change in PO on the right leg even when the left leg is constrained. In all models below, each datapoint represents one test per participant.

**FIGURE 2:**
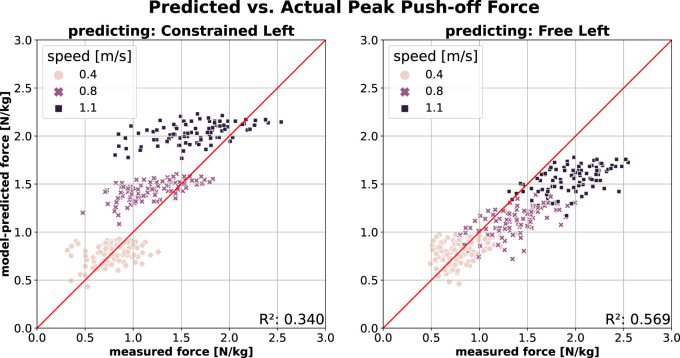
PO prediction across conditions (*Free* → *Constrained*, and *vice versa*) using *Model2* (model inputs: v , $v^{2}$ , f ) (Left) Predicting *Constrained Left* leg using model trained on *Free Left* leg data (Right) Predicting *Free Left* leg using model trained on *Constrained Left* leg data.

Figure 2 shows the PO prediction across the two conditions (from free to constrained and *vice versa*). A model trained on the free walking data (*Free Left*) performs well in predicting PO in constrained walking (*Constrained Left*; Figure 2, left) at slow speed, though its accuracy decreases as speed increases. At higher speeds, the model tends to overestimate measured force, which is reflected in lower ${\text{R}}^{2}$ and higher error (MAE = 0.29 N/kg and MAPE = 28.3%). A similar, albeit less pronounced, trend appears when predicting free walking (*Free Left*) using a model trained on constrained walking data (*Constrained Left*; Figure 2, right). Here, higher walking speeds lead underestimation of the measured push-off force, resulting in denser data distribution. This model performs slightly better overall, as indicated by a higher ${\text{R}}^{2}$ and lower errors (MAE = 0.28 N/kg and MAPE = 20.2%.).

**FIGURE 3:**
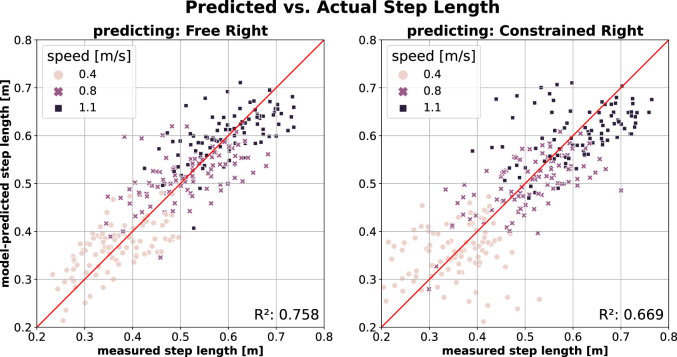
SL prediction of contralateral (i.e., right) leg using model trained on the *Free Left* walking data (*Model2*; model inputs: v , $v^{2}$ , TA ). Right leg is unconstrained in both cases (Left) Predicting right leg’s SL in *Free* walking (Right) Predicting right leg’s SL in *Constrained* walking.

Prediction of SL on the contralateral leg using a model trained on the free walking data (*Free Left*) is shown in Figure 3. The model predicts SL with comparable accuracy in both free walking (*Free Right*; Figure 3, left) and constrained walking (*Constrained Right*; Figure 3, right), though with slightly reduced accuracy in the latter. The difference is reflected by lower ${\text{R}}^{2}$ and higher errors in constrained walking (MAE = 0.048 N/kg and MAPE = 10.4% vs MAE = 0.059 N/kg and MAPE = 13.2%). Unlike the PO prediction, walking speed does not appear to influence SL prediction accuracy. Note that the right leg remains unconstrained in both cases.

**FIGURE 4:**
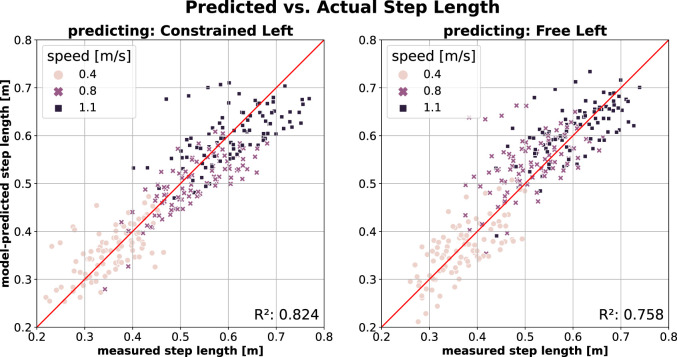
SL prediction across conditions (*Free* → *Constrained*, and *vice versa*) using *Model2* (model inputs: v , $v^{2}$ , TA ) (Left) Predicting *Constrained Left* leg using model trained on the *Free Left* leg data (Right) Predicting *Free Left* leg using model trained on the *Constrained Left* leg data.

Figure 4 illustrates SL prediction across conditions. Predicting SL for the constrained leg (*Constrained Left*) using a model trained on free walking data (*Free Left*; Figure 4, left) yields the best SL prediction performance, as reflected by the highest ${\text{R}}^{2}$ and lowest errors (MAE = 0.056 and MAPE = 13.7%). Predicting SL in the opposite direction–using a model trained on constrained walking data (*Constrained Left*) to predict free walking (*Free Left*; Figure 4, right) – is only slightly less good, with slightly lower (albeit still high) ${\text{R}}^{2}$ and lower errors (MAE = 0.044 and MAPE = 9.4%). Similar to SL predictions within the free walking condition, walking speed does not significantly affect prediction quality in this context.

### 3.2 Spatial and kinetic asymmetries (relative gait metrics)

#### 3.2.1 Peak push-off force (PO) symmetry

As shown in Figure 1, the right leg generates peak push-off force (PO) consistent with predictions from a model trained on free walking, even when participants walked with constraints on their contralateral leg. Conversely, Figure 2 shows that this symmetry does not hold for the left leg during constrained walking, where the peak push-off force (PO) is lower than predicted by a model trained on free walking. This discrepancy suggests a change in PO symmetry, particularly at higher walking speeds.

**FIGURE 5:**
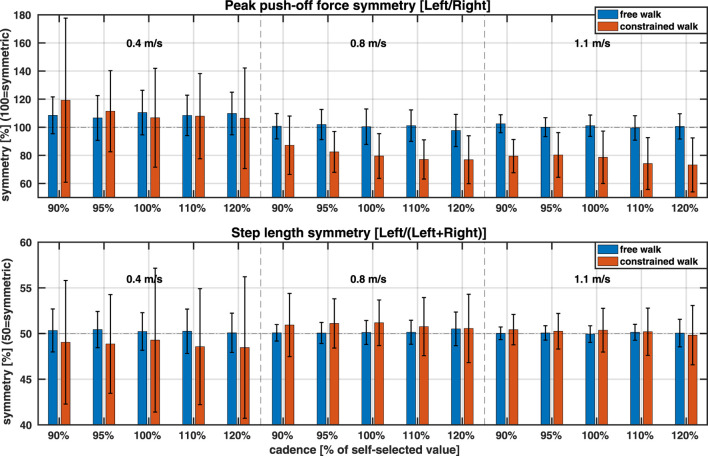
PO and SL symmetry. Data are average across all participants. Three walking speeds are separated by vertical dashed lines, and two conditions (free, constrained) are colour-coded. Horizontal dashed lines indicate perfect symmetry. Note different definitions of the symmetry between PO and SL *(Top)* Peak push-off force symmetry (values below 100 indicate more force by the right leg) *(Bottom)* Step length symmetry (values below 50 indicate shorter left step).

Figure 5 (top) illustrates PO symmetry across conditions, supporting the observations discussed above. During free walking (blue bars), participants on average displayed strong symmetry in their propulsive forces at 0.8 m/s (101.2 ± 11.7% across cadences; mean ± standard deviation) and 1.1 m/s (100.9 ± 7.5% across cadences), with slightly higher PO in their left leg at 0.4 m/s (109.4 ± 14.9% across cadences). Cadence had no statistically significant effect on PO symmetry at any speed; however, there were weak statistically significant differences in PO symmetry across the speeds when averaging across cadences: p= 0.03 between 1.1 and 0.4 m/s and p= 0.04 between 0.8 and 0.4 m/s.

Applying a knee brace on the left leg affected PO symmetry, particularly at 0.8 and 1.1 m/s. At the slowest speed (0.4 m/s), the constraint increased PO symmetry variability, although average PO symmetry remained similar to that during free walking (blue vs red bars; p> 0.99), with an average PO symmetry of 108.2 ± 36.7% across cadences. At 0.8 and 1.1 m/s, PO symmetry on average decreased by approximately 20 percentage points compared to free walking, with the left leg producing less peak propulsive force; across cadences, PO symmetry averaged 79.9 ± 15.8% at 0.8 m/s and 77.7 ± 16.1% at 1.1 m/s. Similar to free walking, cadence had no statistically significant effect on PO symmetry at either of the two speeds. Furthermore, there were no statistically significant differences in PO symmetry between 0.8 and 1.1 m/s during constrained walking, although both speeds were statistically significantly different from 0.4 m/s (strong significance, p< 0.001).

#### 3.2.2 Step length (SL) symmetry

The modelling of SL, depicted in Figures 3, 4, indicated there should be no major asymmetries in SL across speeds in either free or constrained walking. The SL symmetry visualisation in Figure 5 (bottom) confirms this observation. During free walking (blue bars), participants on average walked with strong SL symmetry across all three speeds (50.3 ± 2.1% at 0.4 m/s, 50.1 ± 1.3% at 0.8 m/s, and 50.01 ± 0.9% at 1.1 m/s, averaged across cadences), with cadence having no statistically significant effect on SL symmetry at any speed. Unlike PO symmetry, SL symmetry was unaffected by the knee constraint ( p> 0.59 across all comparisons; notably, the only statistically significant difference, albeit a weak one, was between 0.4 and 0.8 m/s in constrained walking: p= 0.04). Although SL symmetry variability increased across all three speeds, particularly at 0.4 m/s, the overall symmetry remained stable. On average across participants and cadences, SL symmetry during constrained walking remained close to strong symmetry: 49.4 ± 6.7% at 0.4 m/s, 50.2 ± 2.9% at 0.8 m/s, and 50.1 ± 2.4% at 1.1 m/s.

## 4 Discussion

In this study, we investigated how healthy young adults adapt their step length and push-off force across varying speeds and cadences during both unconstrained (free) walking and mechanically constrained (emulated hemiparetic) walking. Using statistical models, we quantified these adaptations in absolute terms and evaluated the predictive capacity of these models using an independent, publicly available dataset. Furthermore, we examined how step length (SL) and peak push-off force (PO) symmetries evolved under different conditions, offering insight into the underlying biomechanical strategies governing gait adjustments.

A key finding of this study is that, despite the imposed asymmetry, participants largely preserved SL symmetry, while PO symmetry was significantly reduced. This suggests that when neural control remains intact, individuals prioritise maintaining a consistent spatial gait pattern at the expense of kinetic symmetry. This contrasts with observations in hemiparetic gait, where step length asymmetries are more pronounced (Hsu et al., 2003; Kim and Eng, 2003), highlighting the distinct biomechanical trade-offs in neurologically intact *versus* impaired populations. Furthermore, we found that the extent of asymmetry adaptation varied across speeds and cadences, demonstrating that functional asymmetry does not induce uniform compensatory strategies but rather context-dependent adjustments.

Another novel contribution is the evaluation of model generalisability. Models trained on free-walking conditions performed well within their respective datasets but showed reduced predictive accuracy when applied to constrained conditions. This underscores the importance of dataset-specific characteristics in gait prediction and suggests that current statistical models may not fully capture the complexity of functional asymmetry adaptations. Our findings highlight the need for hybrid approaches that refine statistical models through broader training datasets and enhanced parameterisation to improve robustness across diverse walking conditions.

### 4.1 Walking dataset

Human gait is often analysed by comparing adaptations between groups, such as speed- or age-matched designs (Dean et al., 2007; Frame et al., 2020). However, these approaches do not account for biomechanical variations independently of speed or age, a limitation that is particularly relevant in patient populations (Ali et al., 2014). The dataset used in this study (Baček et al., 2024) overcomes this by employing a unique within-subject design, isolating gait adaptations due to functional asymmetry from external factors such as muscle weakness. To achieve this, participants walked with a unilateral passive knee brace that restricted left knee flexion, limiting ankle plantarflexion during push-off and prompting compensatory gait adjustments similar to those observed in hemiparetic patients (Baček et al., 2022). Given that higher walking speeds naturally demand greater joint mobility (Mentiplay et al., 2018), we expected increasing speed to pose a greater challenge–an expectation confirmed by our results. While a metronome guided step frequency, participants were allowed small adjustments in step length (SL), step time (ST), and their symmetries, provided their overall stride length and time matched the treadmill-imposed speed.

### 4.2 Statistical modelling of step length (SL) and peak push-off force (PO)

We developed models to predict the absolute amplitude of peak push-off force (PO; a component of the GRF in the anterior-posterior direction) and step length (SL) using gait speed and anthropometric variables. Additionally, cadence was included in the PO model, and trailing limb angle (TA) in the SL model. Previous studies have developed similar models for SL (Park et al., 2021), SL and PO (Yanez et al., 2023), or alternatively, for joint angles and moments (Lelas et al., 2003; Fukuchi et al., 2019b; Fukuchi and Duarte, 2019). However, these studies primarily focused on free walking, aiming to predict gait variables or develop biofeedback targets for clinical applications as alternatives to normative values based on age and sex (Chao et al., 1983; Oberg et al., 1993). In contrast, our study examined the effects of functional asymmetry on SL and PO and explored whether these variables adapt differently across walking speeds.

During free walking, model accuracy was predominantly influenced by speed and speed squared, with anthropometric variables contributing minimally (see Table 1). This finding was consistent for both SL and PO and aligns with results reported by (Yanez et al., 2023). A comparison to (Park et al., 2021) was not possible, as their modelling was limited to a fixed speed. Due to the dataset’s unique design, which included five cadences per speed, our models also benefited from additional inputs–cadence for PO and trailing limb angle for SL–allowing them to account for enforced variations in SL (and consequently, PO) at the same walking speeds.

Models trained on free walking (*Free Left*) demonstrated high accuracy in predicting PO (Figure 1, left) and SL (Figure 3, left) for the contralateral leg (*Free Right*). This suggests that both legs exhibit similar behaviours at a given speed and cadence. Notably, these models also predicted PO (Figure 1, right) and SL (Figure 3, right) for the unconstrained contralateral leg during constrained walking (*Constrained Right*) with only a slight reduction in accuracy, as measured by $R^{2}$ , MAE, and MAPE.

This result is particularly interesting, as it suggests that the unconstrained (right) leg remains largely unaffected by the knee brace applied to the left leg. The most noticeable reduction in prediction accuracy happens at higher speeds in PO, indicating that participants relied more on their unconstrained leg as walking became more challenging. This aligns with findings in hemiparetic patients, where reliance on the unaffected leg increases, albeit independently of speed (Roelker et al., 2019). Conversely, SL prediction accuracy remained stable across speeds, with increased data dispersion across all speeds contributing to minor reductions in prediction quality. Our findings also revealed a lower prediction accuracy for SL compared to PO, consistent with (Yanez et al., 2023), despite their evaluations being based on an independent walking dataset.

We further evaluated model prediction accuracy for PO and SL in the left leg between conditions–transitioning from free to constrained walking and *vice versa*. As shown in Figure 2 (left), the model trained on free walking (*Free Left*) accurately predicted PO for the constrained leg (*Constrained Left*) at slow speeds. However, as speed increased, the model progressively overestimated PO of the constrained (left) leg, indicating that participants produced lower-than-predicted peak push-off forces under constraint. Since the same individuals contributed data for both conditions, this reduction is likely due to the mechanical restriction of the extended knee rather than musculoskeletal limitations such as weakness or impaired neuromuscular control.

This adaptation resembles, though is not identical to, the concept of a propulsive reserve observed in elderly individuals (Franz and Kram, 2012) and hemiparetic patients (Lewek et al., 2018) at their preferred walking speeds. It is plausible that all three groups–hemiparetic patients, elderly individuals, and healthy young adults walking with functional asymmetry (as in this study) – adopt a strategy of generating lower-than-available push-off forces as a preferred compensatory response to impairment, aging, or imposed constraints.

Unlike PO, SL prediction remained unaffected by the addition of the knee constraint (Figure 4). This is unexpected given the critical role that SL modulation plays in maintaining gait stability in the sagittal plane (Hak et al., 2013a; Bruijn and Dieen, 2018); as we’ve shown in (Baček et al., 2025), adaptations in step width are not the source of this. The fact that the knee constraint did not affect absolute SL in the constrained leg compared to the free leg suggests that participants did not rely on SL modulation to adjust stability in the sagittal plane. Instead, they may have prioritised walking energetics over stability. At increasing speeds, humans typically increase both step length and step frequency (Kuo, 2001) to avoid the metabolic penalties associated with disproportionately increasing one over the other (Donelan et al., 2002). If the imposed constraint led to a decrease in stability, participants may have adopted alternative strategies for fore-aft stability modulation (Reimann et al., 2018), or the stability reduction may have remained within tolerable limits, supporting the notion that humans prioritise sufficient rather than maximal gait stability (Hak et al., 2013a).

### 4.3 Spatial (SL) and kinetic (PO) gait symmetries

Healthy young adults typically exhibit kinetic symmetry at walking speeds below 1.5 m/s. This includes symmetry in vertical, braking, and propulsive GRF components (Polk et al., 2017), as well as in propulsive and vertical impulses (Seeley et al., 2008). Our findings align with this: during free walking, participants displayed strong symmetry in peak push-off force at 0.8 and 1.1 m/s, with a slightly higher force on the left leg at 0.4 m/s (Figure 5). Notably, this symmetry persisted across varying step frequencies, even when they walked at non-preferred stride lengths and times (i.e., 90, 95, 110, and 120% of preferred cadence).

Similarly, participants exhibited symmetry in forward (propulsive) and vertical (weight support) impulses, calculated as the ratio of left to right impulses (100% = symmetric). During free walking, weight bearing impulse symmetry showed a slight right-leg dominance (97.65 ± 2.4% across all speeds; see Supplementary Figure A1 in Appendix), while propulsive impulse symmetry showed a consistent left-leg dominance (102.57 ± 14.85% across all speeds; Supplementary Figure A2 in Appendix). Despite minor deviations, these results reinforce the broader notion that healthy adults maintain strong kinetic symmetry across speeds.

Young adults also exhibit strong symmetry in spatial and temporal step parameters. As speed increases, step length (SL) and step time (ST) increase concurrently, likely to optimise metabolic efficiency (Donelan et al., 2002). In the absence of musculoskeletal impairments, humans select an optimal combination of *stride* time and *stride* length at any given speed to minimise metabolic cost (Ralston, 1958) by maintaining equal SLs and STs across both legs, thus avoiding metabolic penalties associated with step asymmetry (Ellis et al., 2013; Stenum and Choi, 2021). Our results align with these findings: during free walking, participants exhibited strong SL symmetry across all speeds and cadences (Figure 5, bottom). Based on the symmetry criteria from (Allen et al., 2011) – where SL is considered symmetric if it falls within the range of 46.5%–53.5% using the same symmetry definition as in this study–all but one participant (at 0.4 m/s) exhibited symmetric SL (see Supplementary Figure A3 (left) in Appendix). Similarly, participants also exhibited strong ST symmetry across speeds and conditions^2^, suggesting that metabolic efficiency remained a key gait priority.

During constrained walking, significant changes in peak push-off (PO) symmetry were observed. At 0.4 m/s, PO symmetry varied considerably across individuals. Notably, three participants shifted from a longer left step (PO symmetry > 100%) to a shorter left step (PO symmetry < 100%), while two showed the opposite trend. At 0.8 and 1.1 m/s, PO symmetry significantly decreased, with nearly all participants showing reduced left-leg propulsion. For instance, at 0.8 m/s, nine participants had higher left push-off forces during free walking, but all except one (Subject 13 in the original dataset) showed lower left push-off under constraint. Similarly, at 1.1 m/s, 13 participants exhibited higher left push-off in free walking, but only Subject 13 retained this pattern in constrained walking. No participants shifted their PO symmetry in the opposite direction. For clarity, individual PO symmetry changes are not shown in Figure 5.

The reduction in left-leg propulsion mirrors findings in stroke patients, where lower paretic propulsion–analogous to the constrained left leg–is compensated by greater non-paretic propulsion, corresponding here to the right leg. With an average PO asymmetry of 80% (Figure 5, top) and a propulsive impulse asymmetry of 72% (see Appendix), our participants resemble *mild* to *moderate* stroke patients classified by (Bowden et al., 2006). In their study, mild stroke patients had a propulsive impulse asymmetry of 49%, moderate patients 36%, and severe patients 16% (calculated as the paretic propulsive impulse divided by the total impulse). When converting our symmetry calculation to match (Bowden et al., 2006), PO asymmetry comes to ≈ 45% and impulse asymmetry to ≈ 42%. The preferred walking speeds of mild to moderate stroke patients in (Bowden et al., 2006) (0.4–1.3 m/s) overlap with the speeds examined here, further reinforcing the potential relevance of these findings to clinical populations.

Functional asymmetry had the least impact on SL symmetry at 1.1 m/s and the highest at 0.4 m/s. At 1.1 m/s, variability in SL symmetry increased slightly. At 0.4 m/s, SL symmetry shifted on average from slightly longer left steps (symmetry > 50%) to slightly shorter left steps (symmetry < 50%), with increased inter-person variability (4.5 percentage points). Anecdotally, participants reported feeling *less stable* and walked with the smallest margin of stability at 0.4 m/s (Baček et al., 2025), potentially explaining these changes. At 0.8 m/s, they slightly increased SL asymmetry, transitioning from near-perfect symmetry in free walking to longer left steps under constraint, with variability doubling at this speed.

Consistent with patient populations (Balasubramanian et al., 2007; Allen et al., 2011), most participants walked with equal or longer steps on the constrained (left) leg. However, unlike the slow speeds typically preferred by stroke patients with higher paretic SL (Allen et al., 2011), our participants with SL asymmetry above 52.5% predominantly walked at 0.8 and 1.1 m/s (7/11 subjects; Supplementary Figure A3). This suggests that increasing speed tends to amplify SL asymmetry, with participants taking longer steps on the constrained leg. Interestingly, deviations in SL symmetry, while remaining within a few percentage points of perfect symmetry (50%), mirrored changes in PO symmetry: participants with reduced constrained-leg propulsion (PO symmetry < 100%) were more likely to take longer left steps. Consequently, the two symmetries correlate (see Supplementary Figure A3 in Appendix), similar to patient population (Balasubramanian et al., 2007).

This suggests a mechanical relationship between step length and propulsive forces, achieved through coordinated control of trunk progression *via* the stance leg and the timing and positioning of the swing leg. For our participants–most of whom took equal or longer left steps despite PO asymmetry–it appears that both mechanisms were engaged. At faster speeds (0.8 and 1.1 m/s), participants took longer steps with their constrained leg. Concurrently, as shown in (Baček et al., 2025), increased right-leg PO in our study facilitated forward CoM progression during the right-leg stance phase, a strategy also employed in patient populations to maintain SL symmetry above 50% despite propulsive deficits.

While propulsive deficits are common in patient populations–regardless of step length asymmetry–forward CoM progression is primarily used when walking with longer paretic steps. Alternative strategies, such as bilateral hip compensation, are often employed to maintain symmetric steps (Allen et al., 2011). Although our study did not analyse joint moments or powers, anecdotal reports of hip fatigue among participants with symmetric SL suggest that they may have adopted similar compensatory mechanisms. Further investigation would be needed to confirm this hypothesis.

### 4.4 Cross-dataset predictions of step length and gait propulsion forces

Statistical gait models, as argued by (Yanez et al., 2023), can serve as individualised biofeedback targets for patients, offering insights into gait pattern deficits. Unlike machine learning models, statistical models provide transparency by revealing not only if, but *how*, independent gait variables influence the dependent outcome. However, model performance reflects both model design and underlying gait dynamics, necessitating multiple cross-dataset predictions to disentangle these factors.

Models developed by (Yanez et al., 2023) were trained on the same F18 dataset as used here^3^. Their model evaluations done using Akaike Information Criterion (AIC) found that gait speed alone is sufficient to predict peak push-off force (PO). Our findings confirm this for both F18 and B24 datasets (Supplementary Table A2). Importantly, we show that including different gait parameters and participant cohorts significantly affects model estimation quality. For example, constrained walking data from B24 and the Elderly cohort from F18 posed significant challenges for model estimation, whereas models trained on B24 free walking performed best. This improvement was most pronounced when accounting for multiple cadences per speed (*Model1* → *Model2*), highlighting the utility of cadence as a model input variable.

Cross-dataset predictions of PO presented herein (B24 → F18, and *vice versa*; Supplementary Figure A5) deliver slightly worse performance as measured by $R^{2}$ compared to the result presented in (Yanez et al., 2023) ( $R^{2}$ = 0.76). However, several important points must be considered. First, our models were trained on a narrower range of speeds and evaluated on fewer data points, likely reducing $R^{2}$ . Second, our cross-dataset predictions separated F18 Young and Elderly groups, which posed distinct modelling challenges (Supplementary Table A2). Third, when models were trained on and tested using the full F18 dataset–similar to (Yanez et al., 2023), i.e., combining Young and Elderly cohorts and using the full range of speeds–the results were comparable: F18 predicting B24 achieved $R^{2}$ values of 0.70 using speed alone (*Model1*) and 0.79 using speed and cadence (*Model2*) but dropped to 0.68 using *Model3*. However, predicting full F18 from B24 resulted in much lower accuracy: $R^{2}$ values of 0.28 for *Model1*, 0.38 for *Model2*, and 0.39 for *Model3* (note that these predictions are not visualised in this paper). When we predicted F18 within the range of speeds present in B24 but still combined Young and Elderly cohorts, the $R^{2}$ only slightly increased (to 0.4 for *Model1* and 0.38 for *Model2* and *Model3*), suggesting that differences in population cohorts have a greater impact than speed range on prediction accuracy.

Similar to PO models, SL model estimation performed well with speed alone as a model input, with AIC evaluations confirming that anthropometric variables add minimal value (Supplementary Table A4). The inclusion of trailing limb angle (TA) as an additional variable (*Model2*) improved SL model estimation in B24, where multiple cadences per speed were tested–an improvement analogous to the role of cadence in PO modelling. These findings align with (Yanez et al., 2023), which also found speed to be a primary predictor for SL while identifying modeling challenges for the Elderly cohort. Unlike PO estimation, constraints had less impact on SL estimation quality. Interestingly, the B24 *Constrained Right* dataset (*Model2* and *Model3*) yielded the most accurate SL models across all conditions, outperforming models trained on F18 data. This suggests that SL dynamics may be less sensitive to constraints than PO, possibly due to compensatory mechanisms that stabilise spatial gait parameters even under altered gait conditions.

SL predictions within the F18 dataset, from Young to Elderly and *vice versa*, performed similarly to cross-dataset predictions in (Yanez et al., 2023), with $R^{2}$ values around 0.7 (*Model1*, Supplementary Figure A6). Cross-dataset predictions from B24 to F18 using *Model1* performed slightly worse when predicting the Elderly cohort ( $R^{2}$ = 0.66) but achieved much better accuracy when predicting the Young cohort ( $R^{2}$ = 0.86; Supplementary Figure A7). Predicting the combined Young and Elderly cohorts from a B24-trained *Model1* yielded intermediate accuracy ( $R^{2}$ = 0.77), aligning with SL prediction in (Yanez et al., 2023) when speed ranges were consistent between training and testing datasets. Notably, the discrepancy in SL prediction accuracy between the Young and Elderly cohorts, absent in PO models, prompted additional analyses with age as a variable.

As shown in Supplementary Figure A8, age did not improve predictions between B24 and F18 Young cohorts (or *vice versa*), as expected due to the similar age distributions. Surprisingly, adding age worsened B24 → F18 Elderly predictions, consistent with other cases where anthropometric variables reduced cross-dataset accuracy (results omitted for space). However, adding age significantly improved predictions from F18 Elderly to B24, suggesting that age plays a more critical role when training models on elderly cohorts, likely reflecting age-related changes in SL dynamics.

Overall, these findings demonstrate that while statistical models can effectively predict SL and PO across datasets, their performance depends on the range and characteristics of the training data. Variations in population dynamics (e.g., age, cadence variability) and experimental design (e.g., speed ranges, constraints) highlight the need for diverse and representative datasets to improve model generalisability. Importantly, the findings also caution against over-reliance on anthropometric variables, which may enhance estimation metrics like AIC but often degrade cross-dataset performance, underscoring the need for rigorous model evaluation.

### 4.5 Limitations

This study provides invaluable insights into human gait adaptations, but it does not come without limitations. First, our cohort comprises only healthy young adults, limiting the generalisability of the presented findings. Although our experimental design intentionally excludes confounding factors common in patient populations, such as reduced strength or neuromuscular control, it is possible that elderly participants or individuals with other impairments would respond differently to imposed functional asymmetry. Second, the passive knee brace used to induce asymmetry and emulate hemiparetic gait does not replicate the biomechanical complexities of pathological conditions, such as stroke, where spasticity and sensory deficits play a significant role in gait adaptations. Third, while the study examines a wide range of walking speeds and cadences, it excludes extreme speeds typically found in clinical populations (e.g., 0.2 m/s and 1.3 m/s), where distinct gait dynamics are known to govern gait patterns. Finally, our focus on short-term gait adaptations within 5 minutes of each condition leaves longer-term compensatory strategies and motor learning unexplored and unknown. Addressing these limitations in future research would enhance the clinical relevance and broader applicability of these findings.

## 5 Conclusion

This study explored how healthy young adults adapt their gait in response to functional asymmetry induced by a unilateral knee constraint, focusing on step length (SL) and push-off force (PO) across varying walking speeds and cadences. The findings reveal distinct adaptation strategies, with participants prioritising spatial over kinetic symmetry, particularly at higher speeds. While SL remained relatively stable across conditions, propulsive force on the constrained leg decreased significantly, prompting compensatory increases in the non-constrained leg’s propulsion. These adaptations align with patterns observed in hemiparetic populations, suggesting shared biomechanical strategies for managing asymmetry.

The results underscore the importance of understanding individual gait adaptations to functional asymmetry, offering valuable insights for rehabilitation strategies in clinical populations. By isolating the effects of a constrained joint on walking dynamics, this study provides a framework for future investigations into the mechanisms underlying asymmetrical gait and highlights the role of biomechanical trade-offs in maintaining efficiency and stability during walking.

Future research should explore how these findings translate to clinical populations. As a next step, our upcoming study–recently approved by the ethics board–will replicate the walking conditions from (Baček et al., 2024) in hemiparetic patients to assess the relevance of the observed compensatory mechanisms in real-world rehabilitation settings. Additionally, this study did not examine joint and limb moment or power analysis, which are crucial for refining these insights and broadening their applicability to diverse patient populations. Investigating longer-term adaptations to functional asymmetry and the role of neuromuscular factors could further enhance gait retraining strategies and support individualised interventions.

## Data Availability

Publicly available datasets were analyzed in this study. This data can be found here: Bacek, T., Sun, M., Liu, H., Chen, Z., Manzie, C., Burdet, E., et al. (2023). A biomechanics and energetics dataset of healthy young adults walking with and without kinematic constraints. figshare doi: https://figshare.com/s/b625dafe53f4f83e21cd519. Fukuchi, C., Fukuchi, R., Duarte, M. (2018): A public data set of overground and treadmill walking kinematics and kinetics of healthy individuals. figshare. Fileset. 10.6084/m9.figshare.5722711.v2.

## Footnotes

1 R-Squared: represents the proportion of variance in the dependent variable predictable from the independent variables. MAE: provides the average magnitude of prediction errors, disregarding direction. MAPE: measures accuracy as a percentage, offering an intuitive view of model performance.

2 Temporal gait aspects, including step time, swing time, and double stance time, as well as their symmetries will be analysed in our future work.

3 Note that (Yanez et al., 2023) included the full range of speeds and combined Young and Elderly data in all models.
